# Serum proteome analysis identifies a potential biomarker for axial psoriatic arthritis

**DOI:** 10.1186/s40001-024-01731-9

**Published:** 2024-03-01

**Authors:** Chaofan Lu, Fan Yang, Shihao He, Hongxia Yu, Qian Wang, Mengtao Li, Xiaofeng Zeng, Xiaomei Leng

**Affiliations:** 1 Department of Rheumatology and Clinical Immunology, Peking Union Medical College Hospital, Chinese Academy of Medical Sciences & Peking Union Medical College, National Clinical Research Center for Dermatologic and Immunologic Diseases (NCRC-DID), Key Laboratory of Rheumatology and Clinical Immunology, Ministry of Education, No1 Shuaifuyuan Wangfujing, Dongcheng District, Beijing, 100730 China; 2 Department of Rheumatology, Guizhou Xingyi People’s Hospital, Xingyi, China

**Keywords:** Arthritis, psoriatic, Proteome, Biomarker, Axial PsA, LC-MS/MS

## Abstract

**Background:**

To identify potential serum biomarkers for differentiating between axial psoriatic arthritis (axPsA) and peripheral psoriatic arthritis (pPsA).

**Methods:**

Serum samples were collected from patients with PsA to create a biomarker discovery cohort and a verification cohort. Patients with PsA were classified into axial or peripheral subtypes based on imaging criteria. Untargeted proteomics technology was used in the discovery phase to screen for biomarkers, and candidate biomarkers were evaluated using enzyme-linked immunosorbent assay (ELISA) in the verification phase.

**Results:**

We identified 45 significantly differentially expressed proteins (DEPs) between axPsA (*n* = 20) and pPsA (*n* = 20) with liquid chromatography-mass spectrometry. Among these DEPs, serum pigment epithelium-derived factor (PEDF) was identified as a candidate biomarker using the Boruta algorithm and lasso regression. Results of ELISA further confirmed that the level of serum PEDF expression was significantly higher in axPsA (*n* = 37) than in pPsA (*n* = 51) at the verification cohort (37.9 ± 10.1 vs. 30.5 ± 8.9 μg/mL, *p* < 0.001). Receiver operating characteristics analysis showed that PEDF had an area under the curve (AUC) of 0.72. Serum PEDF was positively correlated with body mass index and C-reactive protein. Additionally, there was a tendency towards a positive correlation between PEDF and the Bath Ankylosing Spondylitis Disease Activity Index.

**Conclusions:**

This study provided a comprehensive characterization of the proteome in axPsA and pPsA and identified a candidate biomarker, PEDF, that may contribute to early diagnosis for axPsA.

**Supplementary Information:**

The online version contains supplementary material available at 10.1186/s40001-024-01731-9.

## Background

Psoriatic arthritis (PsA) is a musculoskeletal disease that can affect multiple domains, accompanied by other comorbidities such as metabolic syndrome. Some patients with PsA may exhibit axial involvement, referred to as axial PsA (axPsA), while those without axial joint involvement are known as peripheral PsA (pPsA). There is an ongoing controversy regarding whether axial PsA is a distinct disease from axial Spondyloarthritis (axSpA), but multiple studies have pointed out clinical distinctions between axPsA and axSpA [1]. These differences include a less common presence of Inflammatory back pain (IBP), lower rates of HLA-B27 positivity, characteristic axial imaging changes, and so on [2]. IBP, a critical symptom of inflammation in the axial joints for axSpA diagnosis, is found in only 26% of axPsA patients who meet imaging criteria [3]. Secondly, unlike the high positivity rate of HLA-B27 in ankylosing spondylitis [4], the rate of HLA-B27 positivity in patients with PsA is only 20–35% [5, 6], whereas in axPsA this number rises to 43% [7]. All these differences from axSpA may delay the identification of axial involvement in PsA. Conventional synthetic disease-modifying antirheumatic drugs (csDMARDs), which are commonly used in the treatment of pPsA, are considered ineffective for treating axial disease. Guidelines recommend that in patients with predominantly axial disease which is active and has insufficient response to nonsteroidal anti-inflammatory drugs (NSAIDs), therapy with biological disease-modifying antirheumatic drugs (bDMARDs) should be considered [8]. Misdiagnosis of axPsA can result in the progression of axial involvement, leading to joint damage and a significant impact on prognosis [9]. Besides, some patients with PsA only develop axial disease in the late stage of the disease [10, 11], which also implies the need to pay attention to screening during the follow-up period. However, the frequent use of imaging exams undoubtedly increases the radiation and economic burden on patients.

The utilization of reliable biomarkers aids in the early diagnosis of diseases and understanding of the pathogenesis of diseases [12, 13]. Therefore, the discovery of a reliable biomarker in patients with PsA for predicting or identifying involvement of the axial disease would greatly benefit clinicians in prescribing imaging examinations and initiating early use of biologic agents. This, in turn, would improve the prognosis, physical function, and overall quality of life for these patients. Multiple types of samples, such as urine, feces, and saliva can be used as potential samples for research; however, serum and plasma are commonly used due to their ease of collection and stable composition [14]. Techniques for analyzing the serum proteome include mass spectrometry (MS), Multiplex bead- or aptamer-based assays (Slow off-rate modified aptamer scan), and Proximity extension assay (Olink) [15]. Although the latter two techniques offer higher sensitivity, as targeted proteomic techniques, they only monitor the presence or absence of target proteins, whereas MS techniques allow for the hypothesis-free approach with shotgun untargeted MS workflows [16]. Therefore, this study employed untargeted proteomic technology to explore serum biomarkers of axPsA.

## Methods

### Study design and collection of clinical samples

**Fig. 1:**
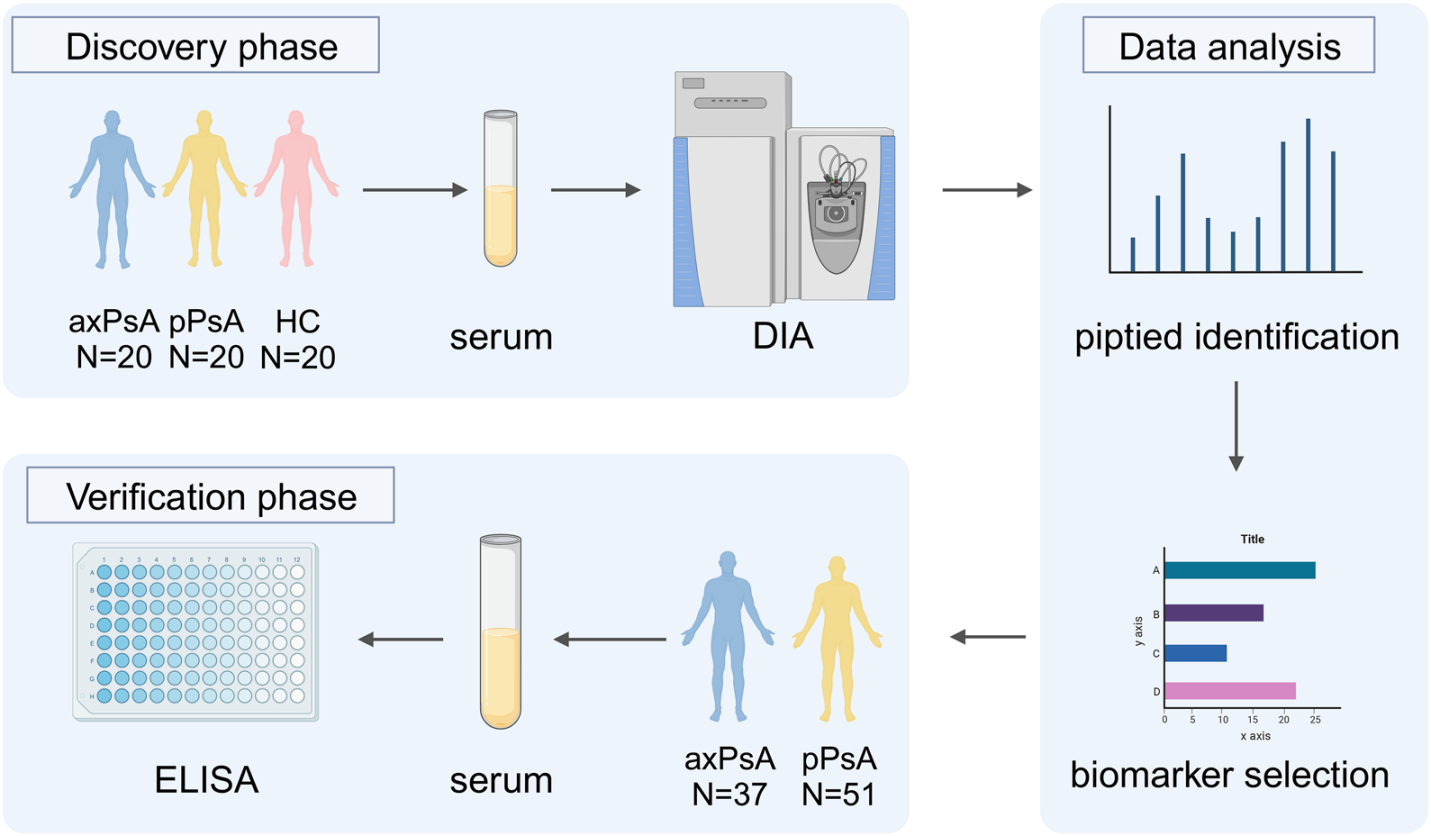
Overview of the experimental workflow. This research is mainly divided into the discovery phase and the verification phase. Liquid chromatography-mass spectrometry (LC-MS/MS) for data-independent acquisition (DIA) was used for biomarker discovery. Random forest, lasso regression, and Boruta algorithm were used to find putative biomarkers that were brought forward for verification with enzyme-linked immunosorbent assay (ELISA). *axPsA* axial psoriatic arthritis, *pPsA* peripheral psoriatic arthritis, *HC* healthy control

The research study was divided into two phases: the discovery phase and the verification phase. The workflow of the study is illustrated in Fig. 1. The study included patients who were diagnosed with PsA based on the Classification Criteria for Psoriatic Arthritis (CASPAR) [17] and also had accessible results of sacroiliac joint computerized tomography (CT) and spinal X-ray, for the formation of the discovery and verification cohorts. The definition of axPsA was based on a previous study [11], which included the presence of New York criteria sacroiliitis (unilateral grade ≥ 3, or bilateral grade ≥ 2 sacroiliitis), and/or≥ 1 marginal/paramarginal syndesmophytes of the cervical or lumbar spine. The grading of sacroiliac arthritis on CT examination was done according to the study by Ye et al. [18]. Patients whose imaging results do not meet the above criteria are classified as negative, indicating pPsA. Clinical parameters and serum samples were collected from a large prospective observational cohort (CREPAR) of patients with PsA at Peking Union Medical College Hospital, which was one of the participating centers in the cohort. Additionally, in the discovery phase, 20 healthy controls matched in terms of age and gender were also enrolled. Serum samples were collected, centrifuged at 3000*g* for 10 min at 4 °C, and stored directly at −80 °C. The study was approved by the ethics committee of the Peking Union Medical College Hospital (K4397). All patients gave their signed informed consent.

### Sample preparation

The ProteoMiner Protein Enrichment Large Capacity Kit (Bio-Rad Laboratories, Hercules, CA, USA) was used for the enrichment of low-abundance proteins, following the manufacturer’s instructions. Briefly, serum was added to spin columns containing the beads and incubated at room temperature for 2 h with constant end-to-end rotation. After washing the columns, the proteins bound to the beads were eluted with the appropriate buffer, divided into aliquots, and stored at −80 °C until use. The protein concentration of each sample was assessed using an assay based on the Bradford method. dl-Dithiothreitol (Sigma-Aldrich) was added (final concentration 10 mM) and the mixture was incubated at 37 °C for 1 h. Iodoacetamide (Sigma-Aldrich) was added (final concentration 10 mM) and the mixture was incubated 45 min in the dark, followed by centrifugation (14000*g*, 15 min). The sample was diluted 4 times by adding 25 mM ammonium bicarbonate buffer. Trypsin (trypsin protein = 1:50) was then added and incubated at 37 ℃ overnight. The tryptic peptides were then acidified with formic acid (FA) to achieve pH < 3 before desalted using Ziptip C18 (Millipore Corp., Billerica, MA, USA). The desalted peptides were then dried under vacuum.

### LC–MS/MS analysis for data-dependent acquisition (DDA)

Quantitative proteomics was conducted by Bio Miao Biological Technology Co, Ltd (Beijing, China). For DDA analysis, we performed on the pooled serum samples. The purified peptide mixtures were separated into 6 fractions using high-pH reversed-phase liquid chromatography and collected. The mass spectrometer used for analysis was the Orbitrap Exploris 480 in combination with the FAIMS PRO and Nanospray Flex Ion Source (Thermo Fisher Scientific, Bremen, Germany). Briefly, the peptide mixture was re-dissolved in buffer A and was loaded on a 25 cm column (150 um inner diameter, packed using ReproSil-Pur C18-AQ 1.9 um silica beads; Beijing Qinglian Biotech Co., Ltd, Beijing, China). Running buffer A was 0.1% FA in water, and running buffer B was 0.1% FA in 80% acetonitrile. Peptides were separated using a gradient starting from 8 to 12% buffer B in 5 min, followed by a gradient from 12 to 30% buffer B over 30 min, and stepped up to 40% buffer B in 9 min. A 16 min wash at 95% buffer B was performed afterward. The total duration of the run was 60 min. MS data were acquired using a DDA method in top speed mode, with the following parameters: full scan resolution 60,000 at *m*/*z* 200, mass range of full mass 350–1500; high-collision dissociation scans with resolution 15,000 at *m*/*z* 200.

### LC–MS/MS analysis for data-independent acquisition (DIA)

Serum peptides were dissolved in a loading buffer containing 0.1% FA and mixed with iRT peptides. The liquid conditions were the same as those of the DDA model. For DIA, the acquisition method consisted of one MS1 scan with a mass range of 350 to 1500 *m*/*z* and a resolution of 60,000; high-collision dissociation scans with resolution 15,000 at *m*/*z* 200. The MS raw data for DIA are publicly available in iProX (accession number: IPX0006903001).

### The identification and quantitation of protein MS data processing

The MS data of the fractionated pools (DDA MS data, 6 fractions) and the single-shot subject samples (DIA MS data) were used to generate a DDA-library and direct-DIA-library respectively, which were computationally merged into a hybrid library in the Spectronaut software (Biognosys, version 15.7). The hybrid spectral library was used to search the MS data of the single-shot samples in the Spectronaut software for final protein identification and quantitation. All searches were performed against the human UniProt reference proteome downloaded in September 2022. Searches used carbamidomethylation as fixed modification and acetylation of the protein N-terminus, and oxidation of methionines as variable modifications. A trypsin/P proteolytic cleavage rule was used, permitting a maximum of two miscleavages and a peptide length of 7–52 amino acids. Default settings were used for other parameters. Spectral library generation stipulated a minimum of three fragments per peptide, and maximally, the six best fragments were included. The identification was performed using a 0.01 false discovery rate threshold on the peptide and protein.

### Verification of biomarker candidates by enzyme-linked immunosorbent assay (ELISA)

The level of serum pigment epithelium-derived factor (PEDF) was measured using commercially available kits according to the manufacturer’s instructions (#ab246535, Abcam, Cambridge, UK). Briefly, 50 µL of assay diluent, human recombinant protein standards, and diluted serum samples were added sequentially to appropriate wells. Subsequently, 50 µL of Antibody Cocktail was added to all wells and incubated at room temperature for 1 h. The wells were then washed three times with washing buffer to remove any unbound detection antibody. Next, 100 µL of TMB Development Solution was added and incubated at room temperature for 10 min. Finally, the reaction was terminated by adding 100 µL of stop solution, and the signal was immediately read at 450 nm.

### Statistics and bioinformatics analyses

For analysis of clinical characteristics, Chi-squared tests or Fisher exact tests were used for categorical variables, while independent samples *T* tests or Mann–Whitney *U* tests were used for continuous variables. Spearman’s rank correlation was utilized to assess the correlation between clinical characteristics and protein levels. A *P* value of< 0.05 was considered statistically significant unless otherwise stated.

For mass spectrometry analysis, protein identification and quantification were conducted using Spectronaut. For quantification, protein intensities were normalized using the median normalization, Log2 transformed, and KNN imputed for comparison between samples. Protein fold changes (on a logarithmic scale) were calculated, and differential expression *P* values were adjusted for multiple testing using the Benjamini–Hochberg method to control the false discovery rate (FDR). Proteins with an adjusted *P* value of < 0.05 were considered significantly differentially expressed between groups. Principal component analysis (PCA) and partial least squares-discriminant analysis (PLS-DA) were used for data dimension reduction. PCA was performed using the ‘stats’ package in R (version 4.2), while PLS-DA was conducted using the ropls package (version 1.30.0). Gene Ontology (GO) annotation, and Kyoto Encyclopedia of Genes and Genomes (KEGG) analysis of differentially expressed proteins (DEPs) were performed using the clusterProfiler package (version 4.6.2). Feature selection was performed with random forest, least absolute shrinkage and selection operator (LASSO) regression, and Boruta algorithm with randomForest package (version 4.7), glmnet package (version 4.1), and Boruta package (version 8.0.0) respectively. Statistical analysis was performed in R (version 4.2) and SPSS (version 22, SPSS Inc., Chicago, USA). Venn diagrams were modified from web-based BioVenn tool [19]. Figures were plotted with R (version 4.2) and GraphPad Prism 8.0.

## Results

### Patient characterization and study design

In the discovery phase, serum samples were collected from patients with axPsA (*n* = 20), pPsA (*n* = 20), and healthy controls (*n* = 20). In the verification phase, we used serum samples from 37 axPsA patients and 51 pPsA patients to evaluate the performance of the candidate protein identified in the discovery phase. Table 1 presents the key demographic and clinical characteristics of all patients. These axPsA and pPsA patients in the discovery cohort had similar levels of peripheral arthritis and inflammation, as indicated by joint counts, C reactive protein (CRP), and erythrocyte sedimentation rate (ESR). Among patients in the verification cohort, those with axPsA had a higher rate of male, smoking and HLA-B27 positivity, higher levels of CRP, and ESR, and lower tender joint count (TJC). Out of the 37 patients with axPsA in the verification cohort, 33 (89.2%) had radiographic sacroiliitis and 18 (48.7%) had syndesmophytes. Specifically, 4 (10.8%) patients had syndesmophytes alone, 18 (48.6%) had sacroiliitis alone, and the remaining patients (40.5%) had both conditions.

**Table 1:** Baseline characteristics of patients with PsA

	Discovery cohort			Verification cohort		
	axPsA (*n* = 20)	pPsA(*n* = 20)	*P*	axPsA (*n* = 37)	pPsA(*n* = 51)	*P*
Gender (male), *n* (%)	14 (70.0)	9 (45.0)	0.110	27 (73.0)	20 (39.2)	0.002
Age, mean (SD)	41.8 (11.9)	40.1 (11.4)	0.647	43.8 (11.1)	42.2 (10.2)	0.696
Comorbidity						
Hypertension, *n* (%)	4 (20.0)	4 (20.0)	1.000	9 (24.3)	9 (17.6)	0.443
Diabetes, *n* (%)	1 (5.0)	0 (0.0)	1.000	3 (8.1)	1 (2.0)	0.305
Hyperlipidemia, *n* (%)	6 (30.0)	5 (25.0)	0.723	8 (21.6)	10 (19.6)	0.817
Ever smoker, *n* (%)	11 (55.0)	6 (30.0)	0.110	20 (54.1)	10 (19.6)	0.001
BMI, mean (SD)	24.7 (5.0)	25.8 (4.3)	0.452	25.1 (21.8, 27.8)^a^	24.3 (22.0, 27.1)^a^	0.826
SJC, median (IQR)	2.0 (0.3, 3.8)	3.5 (0.3, 5.5)	0.317	1.0 (0.0, 4.0)	3.0 (0.0, 6.0)	0.131
TJC, median (IQR)	2.0 (0.3, 6.0)	6.4 (1.8, 10.5)	0.141	1.5 (0.0, 6.0)	4.5 (2.0, 8.8)	0.012
Extra-articular manifestations (current)						
PASI, median (IQR)	2.7 (0.4, 21.4)	1.4 (0.0, 3.4)	0.031	3.0 (0.3, 16.3)	1.3 (0.1, 3.3)	0.081
Enthesitis, *n* (%)	6 (16.2)	14 (27.5)	0.214	6 (16.2)	14 (27.5)	0.304
Dactylitis, *n* (%)	2 (10.0)	3 (15.0)	1.000	5 (13.5)	7 (13.7)	0.977
Extra-articular manifestations (ever)						
Dactylitis, *n* (%)	4 (20.0)	4 (20.0)	1.000	10 (27.0)	12 (23.5)	0.708
Enthesitis, *n* (%)	11 (55.0)	12 (60.0)	1.000	21 (56.8)	31 (60.8)	0.704
IBD, *n* (%)	0 (0.0)	0 (0.0)	/	1 (2.7)	2 (3.9)	1.000
Uveitis, *n* (%)	1 (5.0)	1 (5.0)	1.000	4 (10.8)	1 (2.0)	0.157
CRP, median (IQR)	9.8 (4.8, 33.2)	4.5 (1.1, 28.9)	0.176	8.4 (3.8, 26.8)	2.7 (0.9, 13.0)	0.005
ESR, median (IQR)	27.5 (12.0, 63.3)	12 (3.5, 27.7)	0.085	14 (10.0, 38.0)	11.0 (4.3, 22.5)	0.043
HLA-B27 (+), *n* (%)	8 (40.0)	2 (10.0)	0.028	12 (38.7)	4 (8.7)	0.001
DAPSA, median (IQR)	19.6 (10.4, 29.7)	20.5 (10.2, 27.9)	0.968	14.9 (8.4, 27.3)	18.3 (10.3, 26.2)	0.455
DAS28-CRP, mean (SD)	3.9 (1.9)	3.5 (1.4)	0.610	3.3 (1.4)	3.3 (1.2)	0.869
DAS28-ESR, mean (SD)	3.6 (1.4)	3.4 (1.2)	0.472	3.4 (1.7)	3.4 (1.3)	0.887

*axPsA* axial psoriatic arthritis, *pPsA* peripheral psoriatic arthritis, *BMI* body mass index, *SJC* swollen joint count, *TJC* tender joint count, *PASI* psoriasis area and severity index, *IBD* inflammatory bowel disease, *CRP* C-reactive protein, *ESR* erythrocyte sedimentation rate, *DAPSA* disease activity index for psoriatic arthritis, *DAS28* disease activity score 28

^a^Data were described with median (IQR)

### Unbiased LC–MS/MS-based protein analysis

**Fig. 2:**
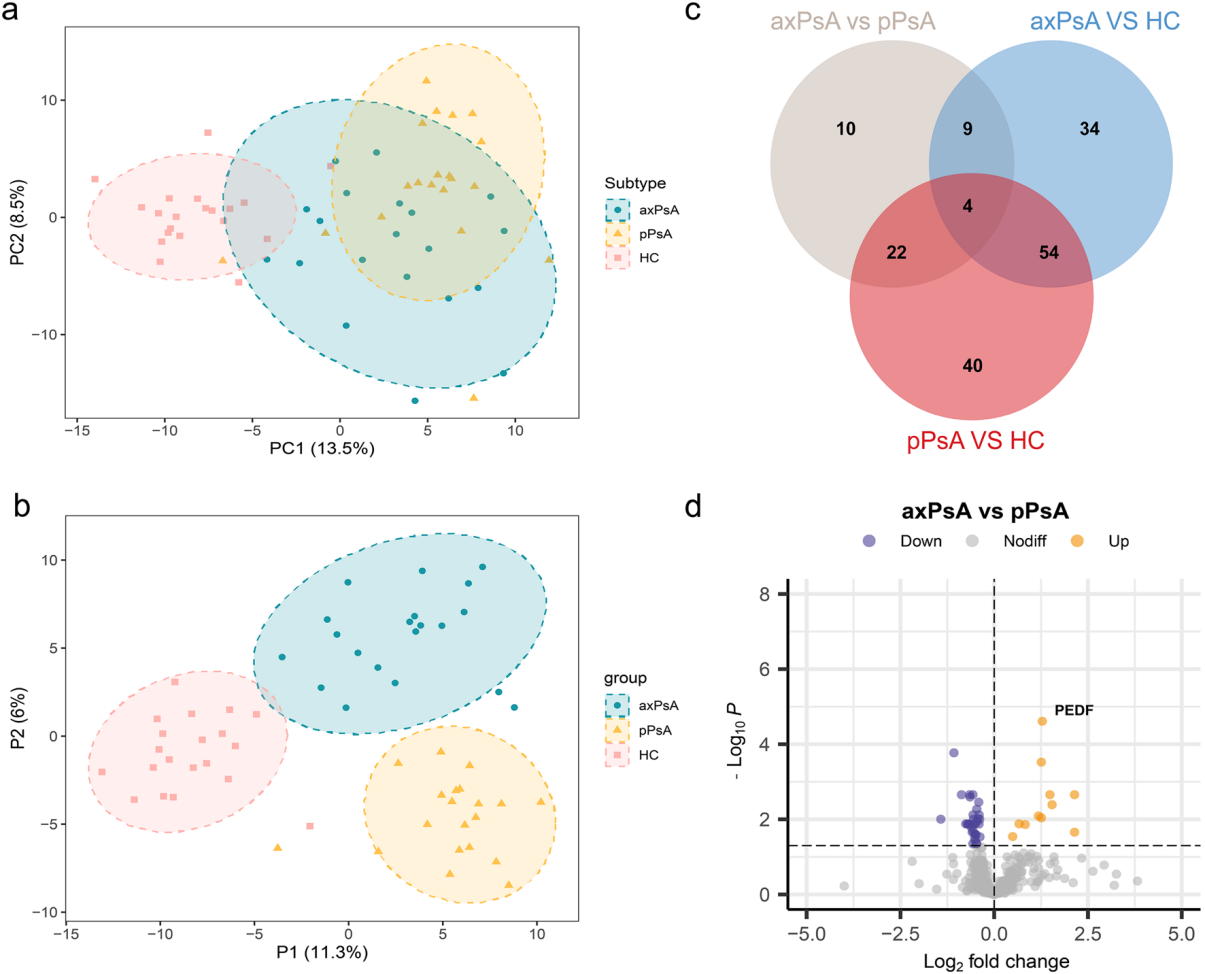
Unbiased L-MS/MS-based protein analysis. **a** PCA score plot of the serum samples of the discovery cohort. **b **PLS-DA score plot of the serum samples of the discovery cohort. **c** This Venn diagram shows the number of DEPs found in the pairwise comparison among the three groups. **d** The volcano plot shows the DEPs between axPsA and pPsA. PEDF was the top-upregulated DEP according to the p-value. *axPsA* axial psoriatic arthritis, *pPsA* peripheral psoriatic arthritis, *HC* healthy control, *DEPs* differentially expressed proteins, *PEDF* pigment epithelium-derived factor

The identified proteins underwent dimensionality reduction analysis. Figure 1a shows the PCA score plot of identified proteins between different groups. The distinction between HC and all PsA patients (pPsA and axPsA) was clear, but the distance between pPsA and axPsA group was not clear. These findings indicated significant proteome changes in patients with PsA compared with HC. Considering that the application of PCA only reveals group structure when within-group variation is sufficiently less than between-group variation. PLS-DA, a kind of supervised form of discriminant analysis, which can preset classifications and add grouping variables to further strengthen the differences between groups was used. The results of PLS-DA revealed significant differences among the three sample groups (R2Y = 0.802, Q2 = 0.7) (Fig. 2b).

We identified a total of 130 DEPs when comparing healthy controls and all patients with PsA. When comparing axPsA and HC, pPsA and HC, and axPsA and pPsA, we identified 101, 120, and 45 DEPs, respectively. The overlap between these DEP group sets was analyzed with the Venn diagram, which is illustrated in Fig. 2c. Among these 45 DEPs between axPsA and pPsA, 11 proteins were found to be up-regulated, while 34 proteins were down-regulated in axPsA. A volcano plot was used to visualize the changes in protein expression between axPsA and pPsA, as shown in Fig. 2d. Among these proteins, PEDF was found to be the top significantly upregulated protein in axPsA, based on the adjusted *P* value.

Using the bioinformatics method, we conducted an analysis of the DEPs between axPsA and pPsA. The results of the GO analysis indicated that the DEPs were associated with biological processes related to innate immunity like complement, coagulation, and the regulation of proteolytic activity. Furthermore, these DEPs were involved in the regulation of enzyme activity, which was identified as the most important molecular function. KEGG analysis showed that these DEPs were mainly involved in the complement pathway and hemostasis (Additional file 1: Figure S1).

### Identification of candidate biomarkers

**Fig. 3:**
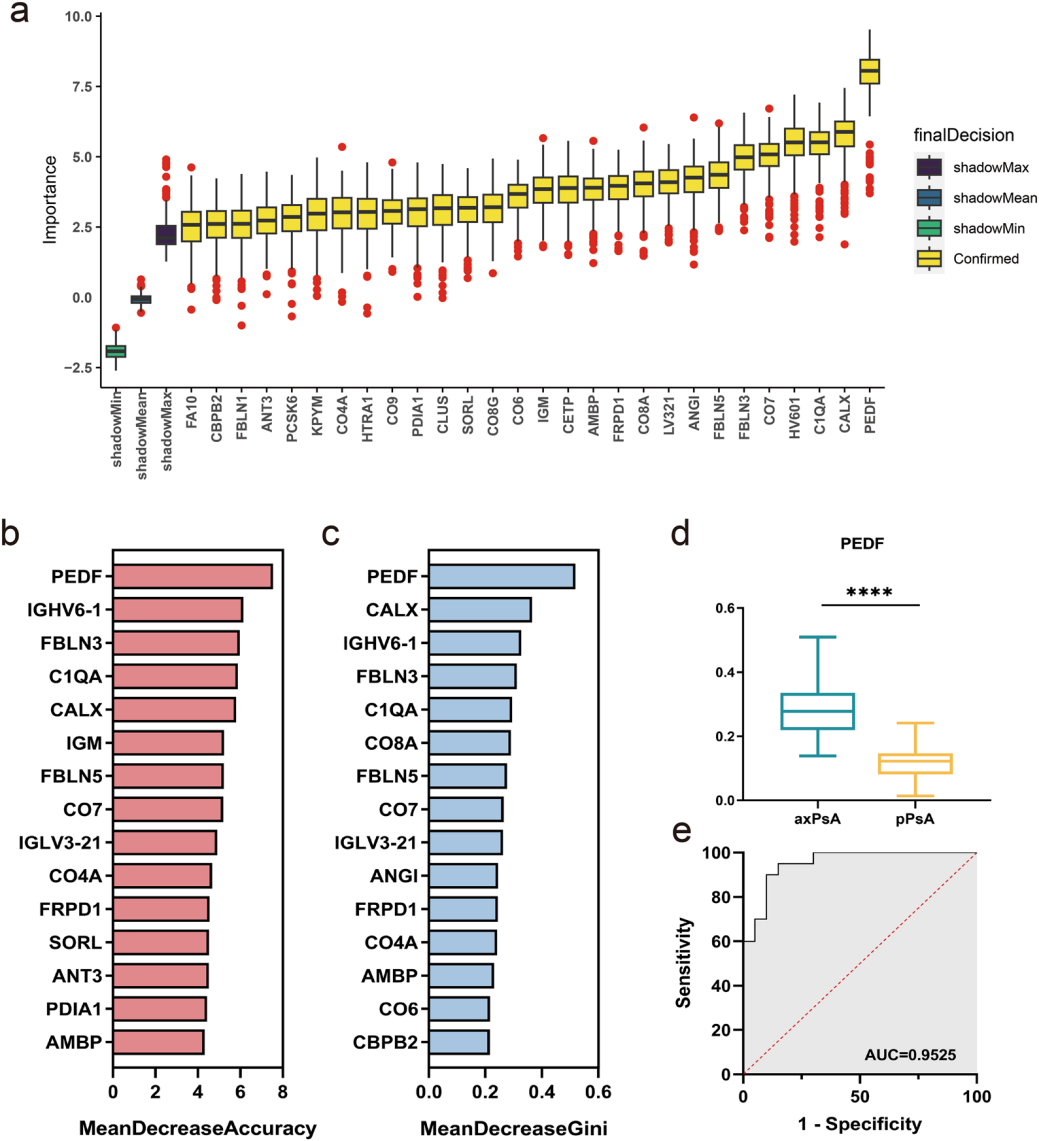
Identification of candidate biomarkers based on MS data from the discovery cohort. **a** Feature selection based on the Buruta algorithm; Feature ordering based on mean decrease accuracy (**b**) and mean decrease gini (**c**) in random forest model. PEDF was the top rank DEP; **d** quantitative analysis of serum PEDF levels in two groups using mass spectrometry data (****p < 0.001). The intensity of PEDF was normalized. **e** Receiver operating characteristic curve analysis of candidate biomarkers for axPsA vs. pPsA based on quantitative analysis of mass spectrometry data. *PEDF* pigment epithelium-derived factor, *DEPs* differentially expressed proteins, *axPsA* axial psoriatic arthritis, *pPsA* peripheral psoriatic arthritis

To better identify clinically available biomarkers, we employed various approaches to select candidate biomarkers. Boruta analyses confirmed 28 biomarkers that are important for identifying patients. Random Forest (RF) analyses were conducted to objectively evaluate the importance of serum proteins, and the top ten proteins are displayed in Fig.3b, c. Additionally, LASSO regression selected 7 DEPs. Among these candidate biomarkers, PEDF appeared in all lists and ranked first in RF. Therefore, we selected PEDF as a potential marker for further verification. Based on quantitative analysis using mass spectrometry data, we observed a significant upregulation of PEDF in axPsA compared to pPsA. Receiver operating characteristic (ROC) analyses of PEDF yielded an area under the curve (AUC) value of 0.925.

### ELISA verification of MS-identified biomarkers

**Fig. 4:**
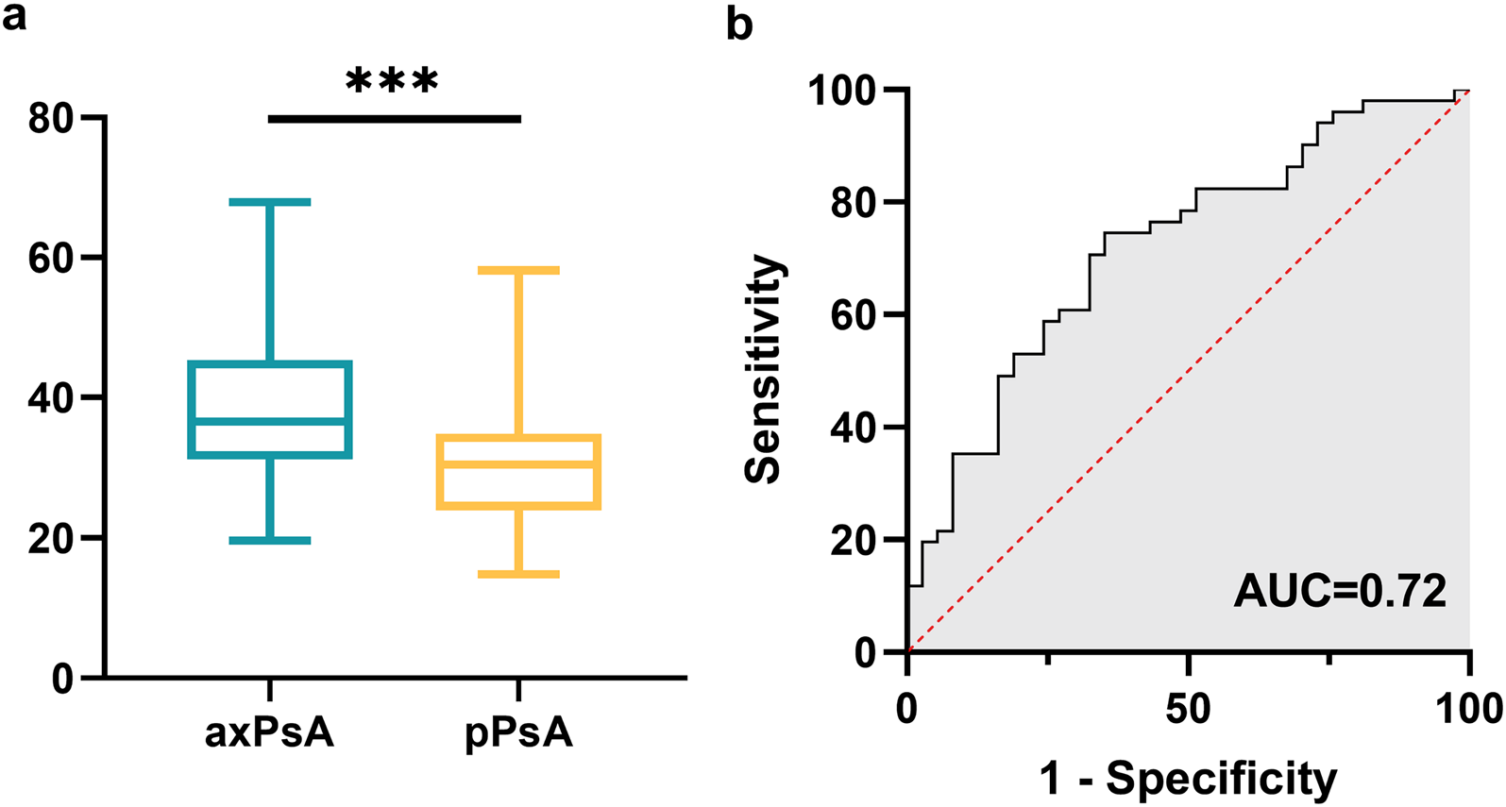
The level of PEDF in the patients in the verification cohort. **a** The comparison of serum PEDF between axPsA and pPsA with ELISA. (***p < 0.001). **b** Receiver operating characteristic curve analysis of candidate biomarkers for axPsA vs. pPsA. *PEDF* pigment epithelium-derived factor, *axPsA* axial psoriatic arthritis, *pPsA* peripheral psoriatic arthritis

**Fig. 5:**
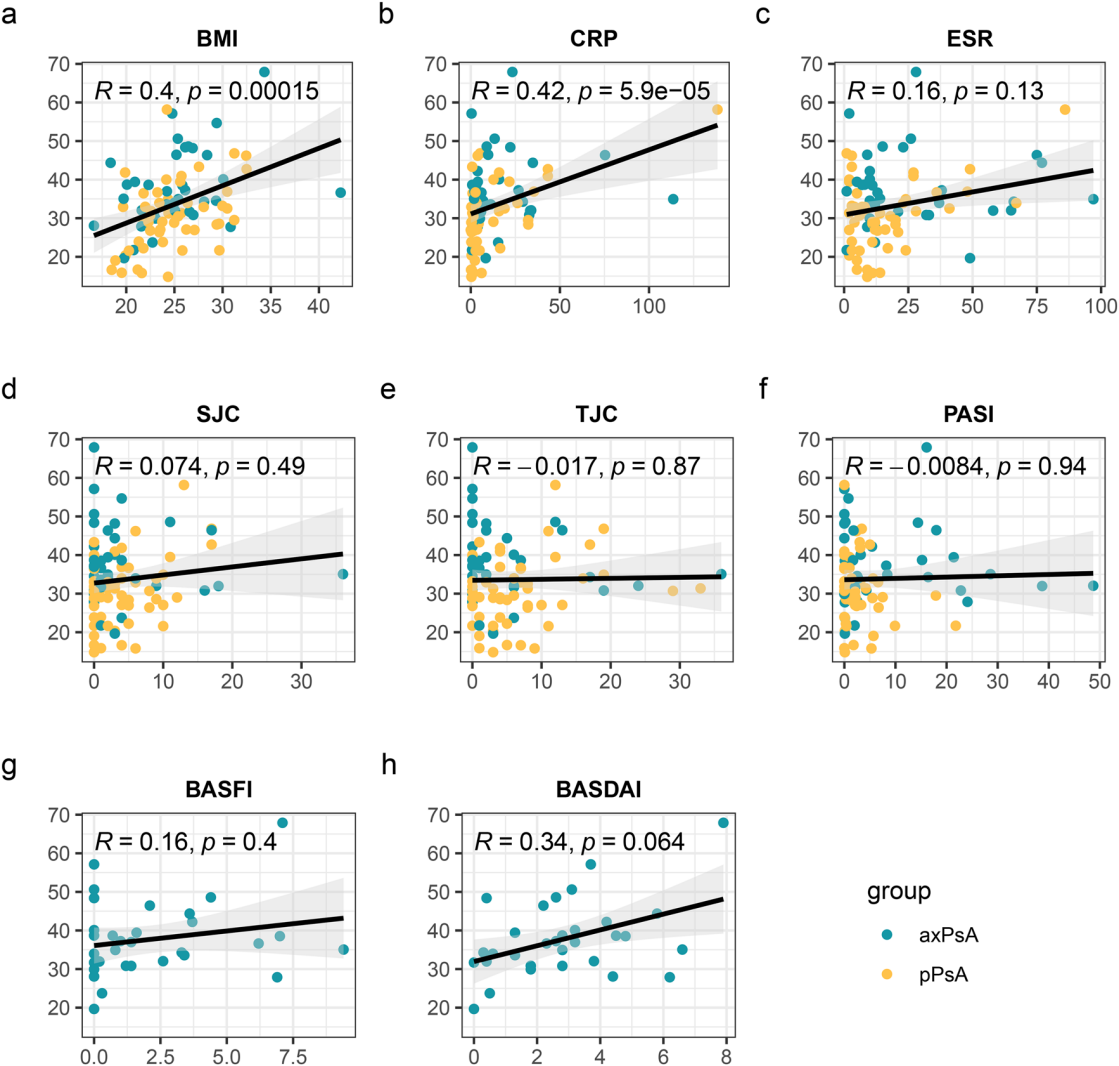
Correlation analysis between the serum level of PEDF and major disease manifestations. The correlation between BMI (**a**), CRP (**b**), ESR (**c**), SJC (**d**), TJC (**e**), PASI (**f**), BASFI (**g**), BASDAI (**h**), and serum PEDF. The data of BASDAI and BASFI was only available in patients with axPsA. *BMI* body mass index, *CRP* C reactive protein *ESR* erythrocyte sedimentation rate, *SJC* swollen joints count, *TJC* tender joints count, *PASI* Psoriasis Area and Severity Index, *BASFI* Bath AS Functional Index, *BASDAI* Bath Ankylosing Spondylitis Disease Activity Index

To further investigate the expression of PEDF in the serum of patients with axPsA and pPsA, we detected the level of PEDF in 37 patients with axPsA, and 51 patients with pPsA by ELISA in the verification phase. As shown in Fig. 4, PEDF expression was significantly higher in axPsA compared with pPsA (37.9 ± 10.1 vs. 30.5 ± 8.9 μg/mL, *p* < 0.001), the AUC score was 0.72 (95%CI 0.61–0.83). PEDF, BMI, and the clinical variables with significant differences in univariate analysis were included in the multivariate analysis. The results showed that PEDF remained significantly elevated in axPsA patients (*P* = 0.017, Additional file 1: Table S1). There were no significant differences observed in the level of PEDF among axPsA patients with different imaging types (Additional file 1: Figure S2). We performed a correlation analysis between the serum levels of PEDF and major disease manifestations in patients with PsA. The results showed that serum PEDF was positively correlated with BMI (*r* = 0.4, *P* < 0.001) and CRP (*r* = 0.42, *P* < 0.001). Additionally, a noticeable trend towards a positive correlation between PEDF and Bath Ankylosing Spondylitis Disease Activity Index (BASDAI) was observed (*r* = 0.36, *P* = 0.064). There were no significant correlations found between serum PEDF levels and swollen joint count (SJC), TJC, and psoriasis area and severity index (PASI) (Fig. 5).

## Discussion

In recent years, there has been a growing interest in studying axPsA. Several studies have identified distinct characteristics that differentiate axPsA from axSpA, which may delay diagnosis and treatment [20, 21]. While reliable biomarkers can aid clinicians in prescribing more targeted imaging tests and identifying asymptomatic individuals with axial involvement, it is imperative to ensure that these patients receive timely diagnoses and appropriate treatments, such as interleukin-17A inhibitors. These inhibitors have been proven to delay radiographic progression and prevent loss of function [22]. Thus, this study utilized mass spectrometry technology to explore biomarkers capable of distinguishing between axPsA and pPsA and confirmed the dependability of serum PEDF as a potential biomarker.

As we all know, the efficacy of a biomarker is related to the definition of the disease. An important initiative in this field is the Axial Involvement in Psoriatic Arthritis cohort (AXIS) study, which aims to establish classification criteria for axPsA [23]. Considering the objectivity of imaging examinations and the poor sensitivity and specificity of existing IBP criteria in patients with axPsA [24], we refer to the imaging criteria used in previous studies. For the identification of axPsA in our study, we employed imaging criteria from previous research, including the New York criteria for sacroiliitis and/or syndesmophyte of the spine [11]. Given the high accessibility of sacroiliac joint CT in our cohort, a large number of patients had access to this imaging data. Additionally, several studies have shown that CT demonstrates superior diagnostic accuracy for axSpA [25]. Another study comparing magnetic resonance imaging and CT evaluations of axial lesions in the sacroiliac joint also indicated that CT has excellent specificity and good sensitivity [18]. Thus, we opted to use CT to evaluate the lesions in the sacroiliac joint. Based on the definition provided, we have identified PEDF as a potential biomarker.

PEDF, also known as pigment epithelium-derived factor, is a 50 kDa secreted glycoprotein belonging to the non-inhibitory serpin family group. It was originally identified as an active component in the culture medium of human fetal retinal pigment epithelial cells, and it was found to have the potential to induce the differentiation of Y79 retinoblastoma tumor cells into non-proliferating neurons [26]. Subsequent research revealed that PEDF has diverse functions, including the induction of cell differentiation and neuronal protection, as well as anti-angiogenic and anti-tumor effects [27]. Moreover, there is increasing recognition of the role of PEDF in maintaining bone homeostasis [28].

PEDF upregulates the expression of vascular endothelial growth factor (VEGF) in human mesenchymal stem cells, and this upregulation is mediated through the ERK signaling pathway. VEGF derived from osteoblasts plays a crucial role in various stages of bone repair and promotes osteogenesis, indirectly suggesting that PEDF may have a role in bone mineralization [29]. Another study also confirmed that PEDF activates the ERK and AKT signaling pathways in mesenchymal stem cells and induces the expression of osteoblast-related genes, thereby participating in the differentiation of mesenchymal stem cells into osteoblasts [30]. Sclerostin, which is secreted by osteocyte dendrites and acts as a potent inhibitor of bone formation [31], can be inhibited by exogenous PEDF supplementation [32]. In addition to its role in promoting bone formation, PEDF may also potentially inhibit bone resorption [33]. The coexistence of bone erosions and abnormal new bone formation (osteophytes, enthesophytes) is a distinguishing feature of PsA [34]. The elevated serum PEDF levels in patients with axPsA imply that osteogenesis is implicated in the pathogenesis of the axial disease, which aligns with the presence of sacroiliac joint involvement and the formation of syndesmophytes or large paramarginal syndesmophytes in axPsA.

In the analysis regarding the correlation between PEDF and clinical data, we observed a positive association between CRP, BMI, and PEDF. Based on previous studies, there have been similar findings indicating a connection between PEDF and CRP in patients with coronary heart disease, polycystic ovary syndrome, diabetes mellitus, and chronic obstructive pulmonary disease [35–38]. It might be possible in the future to consider PEDF as a potential marker for evaluating the level of inflammation in patients with PsA. CRP indicates the level of systemic inflammation in PsA, and any affected clinical domain may contribute to the increase in inflammation. Therefore, we also examined the relationship between serum PEDF levels and different clinical domains. The results show that there is no correlation between PEDF and PASI, which reflects the severity of skin lesions, as well as SJC and TJC, which reflect the activity of peripheral arthritis. However, there is a positive correlation trend between PEDF and BASDAI, which reflects the activity of axial involvement, although it did not reach statistical significance. This may indicate that the increased levels of PEDF in axPsA patients are associated with axial involvement rather than other affected clinical domains. It also suggests the potential of PEDF in aiding the assessment of disease activity in axial diseases. However, further research is needed to confirm this possibility.

To the best of our knowledge, this study was the first exploration of a biomarker for the axPsA subtype using proteome-based analysis. We have successfully identified serum PEDF as a potential biomarker. However, we acknowledge that there are limitations to this experiment. Firstly, it is important to note that this study was conducted at a single center, which may limit the generalizability of the findings. The sample size of the study was also relatively small. To increase the reliability and validity of future studies, it would be beneficial to include larger sample sizes and conduct multicenter validation studies. Secondly, although we observed higher levels of PEDF in axial PsA during the verification stage, its ability to differentiate patients decreased compared to the discovery phase, with the AUC dropping from 0.93 to 0.72. This may be because the two groups of patients were matched during the discovery phase and efforts were made to minimize confounding factors, while the verification stage aligned better with the clinical environment. In this case, by expanding from the analysis of an individual protein biomarker to protein panels, more effective tools can be developed to guide diagnosis and therapeutic choices [39]. A suite of machine learning techniques, such as logistic regression, random forests, and support vector machines can be used in the identification of a multivariate biomarker panel [40]. Thirdly, the patients included in this study were not treatment-naive, and the therapeutic medications may have influenced the outcomes. However, the unselected patients in this study align more closely with the real clinical environment and are more conducive to clinical applicability.

## Conclusions

In conclusion, we utilized mass spectrometry to analyze the serum proteome in patients with axPsA and pPsA, and identified several DEPs between the two groups. AxPsA and pPsA have distinct serum protein profiles that can be used as biomarkers to discriminate between them. Among these proteins, PEDF showed promise as a potential biomarker, and its validity was confirmed using ELISA in a larger verification cohort. However, further validation is still needed in patients from an expanded or independent cohort before it becomes a truly reliable marker for clinical practice. Additionally, considering the clinical heterogeneity and potential comorbidities in patients with PsA, a biomarker panel with multiple proteins may be a more ideal diagnostic tool.

### Supplementary Information

**Additional file 1:Figure S1.** Enrichment analysis of DEPs between axPsA and pPsA.(A) Gene Ontology (GO) classification of the DEPs. The top 10 enriched terms in the Biological Process (BP), Cellular Component (CC), and Molecular Function (MF) are listed. (B) Kyoto Encyclopedia of Genes and Genomes (KEGG) pathway analysis of the DEPs. The top 10 enriched pathways are listed. axPsA, axial psoriatic arthritis; pPsA, peripheral psoriatic arthritis; DEPs, differentially expressed proteins. **Table S1.** Multivariate logistic regression analysis for clinical characteristics and serum PEDF between axPsA and pPsA.

## Data Availability

The mass spectrometry proteomics data have been deposited to the ProteomeXchange Consortium via the iProX partner repository with the dataset identifier PXD044598 (Accession Number: IPX0006903001).

## Abbreviations

AUC: Area under the curve
axPsA: Axial psoriatic arthritis
axSpA: Axial spondyloarthritis
BASDAI: Bath ankylosing spondylitis disease activity index
BASFI: Bath ankylosing spondylitis functional index
bDMARDs: Biological disease-modifying antirheumatic drugs
BMI: Body mass index
CASPAR: Classification criteria for psoriatic arthritis
CRP: C-reactive protein
csDMRADs: Conventional synthetic disease-modifying antirheumatic drugs
CT: Computerized tomography
DAPSA: Disease activity index for psoriatic arthritis
DAS28: Disease activity score 28
DDA: Data-dependent acquisition
DEPs: Differentially expressed proteins
DIA: Data-independent acquisition
ELISA: Enzyme-linked immunosorbent assay
ESR: Erythrocyte sedimentation rate
FA: Formic acid
GO: Gene ontology
HC: Healthy control
LASSO: Least absolute shrinkage and selection operator
IBD: Inflammatory bowel disease
KEGG: Kyoto encyclopedia of genes and genomes
MS: Mass spectrometry
NSAIDs: Nonsteroidal anti-inflammatory drugs
PASI: Psoriasis area and severity index
PCA: Principal component analysis
PEDF: Pigment epithelium-derived factor
PLS-DA: Pleast squares-discriminant analysis
pPsA: Peripheral psoriatic arthritis
PsA: Psoriatic arthritis
ROC: Operating characteristic
SJC: Swollen joint count
TJC: Tender joint count
VEGF: Vascular endothelial growth factor
