# Supplementary material for: Serum proteome analysis identifies a potential biomarker for axial psoriatic arthritis
Source: Eur J Med Res. 2024 Mar 1;29:146. doi: 10.1186/s40001-024-01731-9 (PMC10908212; doi:10.1186/s40001-024-01731-9)
Supplement: Supplementary file 1 — Additional file 1:Figure S1. Enrichment analysis of DEPs between axPsA and pPsA.(A) Gene Ontology (GO) classification of the DEPs. The top 10 enriched terms in the Biological Process (BP), Cellular Component (CC), and Molecular Function (MF) are listed. (B) Kyoto Encyclopedia of Genes and Genomes (KEGG) pathway analysis of the DEPs. The top 10 enriched pathways are listed. axPsA, axial psoriatic arthritis; pPsA, peripheral psoriatic arthritis; DEPs, differentially expressed proteins. Table S1. Multivariate logistic regression analysis for clinical characteristics and serum PEDF between axPsA and pPsA. [file 40001_2024_1731_MOESM1_ESM.docx]

**Serum proteome analysis identifies a potential biomarker for axial psoriatic arthritis**

**Journal: European Journal of Medical Research**

**Authors**: Chaofan Lu, Fan Yang, Shihao He, Hongxia Yu, Qian Wang, Mengtao Li, Xiaofeng Zeng, Xiaomei Leng

**Corresponding Author:** Xiaofeng Zeng and Xiaomei Leng

**Email**: [zengxfpumc@163.com](mailto:zengxfpumc@163.com); [lpumch@126.com](mailto:lpumch@126.com).

**This PDF file includes:**

Supplementary Figures (Fig. S1 to S2)

Supplementary Table

**Additional Figures**
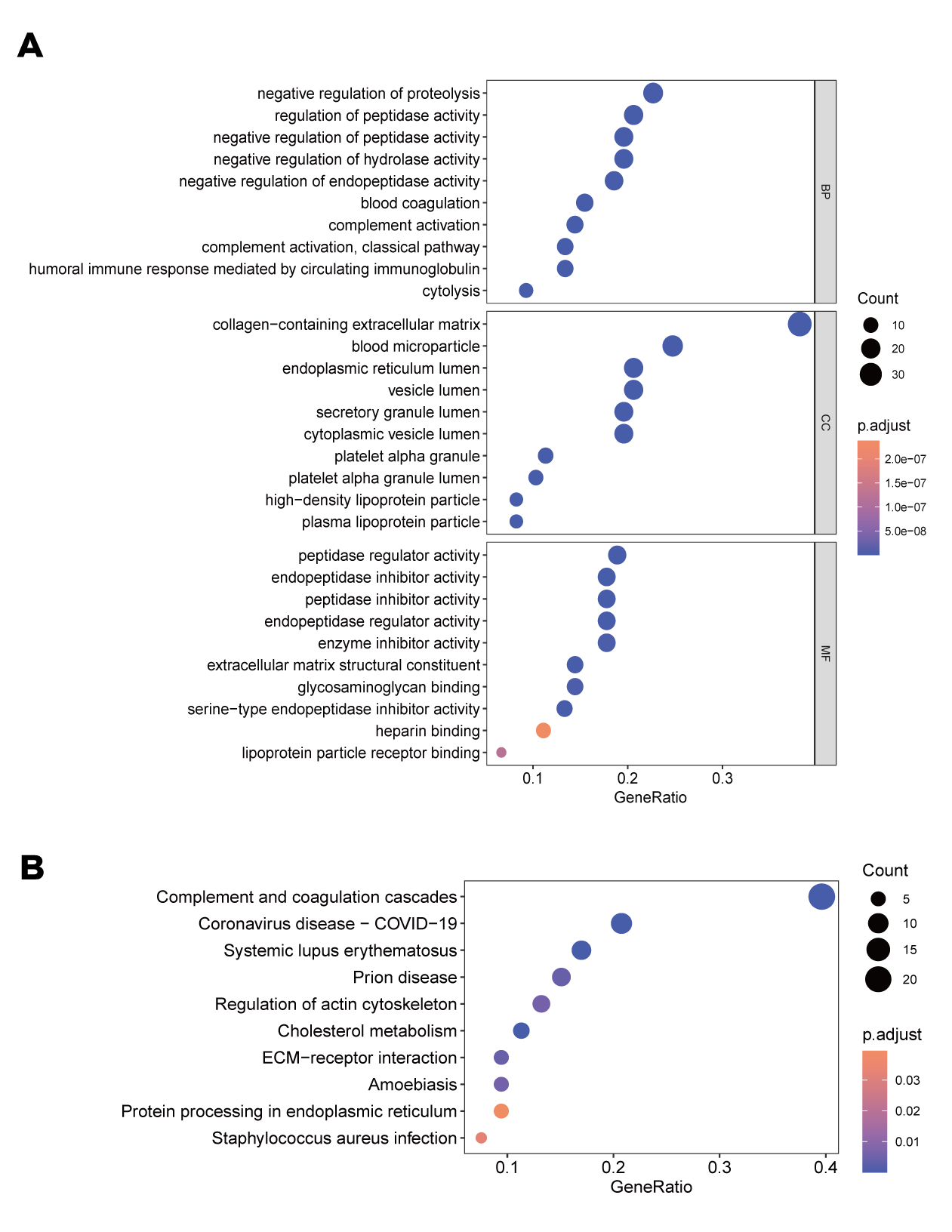


**Fig.S1** Enrichment analysis of DEPs between axPsA and pPsA

**(A)** Gene Ontology (GO) classification of the DEPs. The top 10 enriched terms in the Biological Process (BP), Cellular Component (CC), and Molecular Function (MF) are listed. **(B)** Kyoto Encyclopedia of Genes and Genomes (KEGG) pathway analysis of the DEPs. The top 10 enriched pathways are listed.

Abbreviations: axPsA, axial psoriatic arthritis; pPsA, peripheral psoriatic arthritis; DEPs, differentially expressed proteins.

**
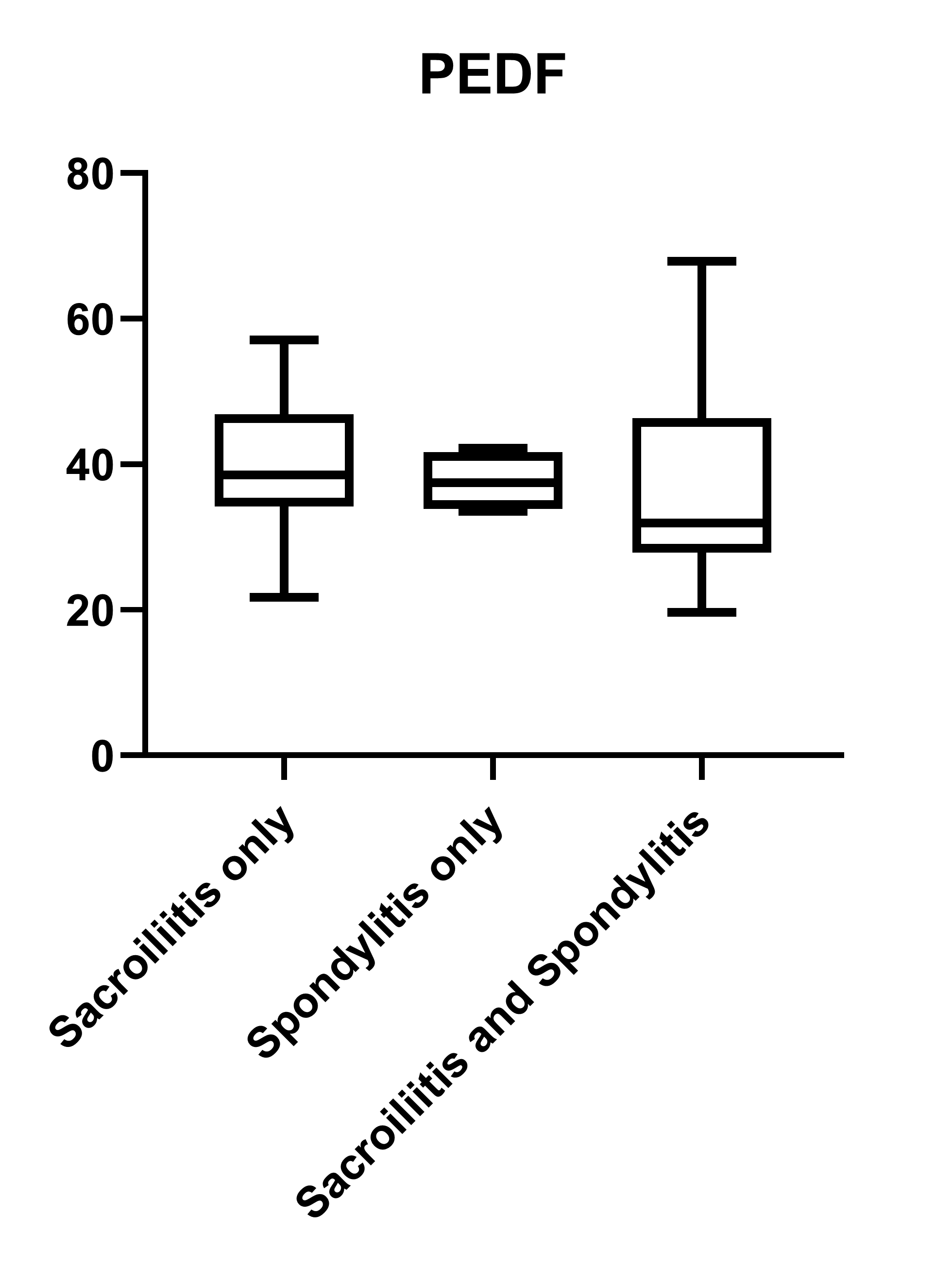
Fig.S2** The level between axPsA patients with different imaging changes type.

**Additional Table**

Table S1. Multivariate logistic regression analysis for clinical characteristics and serum PEDF between axPsA and pPsA.

|  | P | β | Std. Error | OR | 95% CI |
| --- | --- | --- | --- | --- | --- |
| PEDF | 0.017 | 0.093 | 0.041 | 1.097 | 1.017~1.184 |
| Ever smoker | 0.043 | 1.374 | 0.680 | 3.950 | 1.041~14.990 |
| TJC | 0.120 | -0.086 | 0.044 | 0.934 | 0.857~1.018 |
| CRP | 0.041 | 0.022 | 0.063 | 0.960 | 0.920~1.002 |
| ESR | 0.015 | 0.060 | 0.025 | 1.062 | 1.012~1.115 |
| BMI | 0.092 | -0.129 | 0.076 | 0.879 | 0.757~1.021 |
| Male | 0.180 | 0.960 | 0.716 | 2.612 | 0.642~10.631 |
| HLA-B27(+) | 0.070 | 1.521 | 0.839 | 4.575 | 0.883~23.695 |

Abbreviations: axPsA, axial psoriatic arthritis; pPsA, peripheral psoriatic arthritis; TJC, tender joint count; CRP, C-reactive protein; ESR, erythrocyte sedimentation rate; BMI, body mass index; PEDF, pigment epithelium-derived factor.
